# Intracerebral hemorrhage and carotid-cavernous fistulas: a rare connection

**DOI:** 10.1007/s00234-026-03933-w

**Published:** 2026-03-06

**Authors:** Christian Gronemann, Sarah Panahabadi, Julia Layer, Felix J Bode, Franziska Dorn

**Affiliations:** 1 Department of Neuroradiology, University Hospital Bonn, Bonn, Germany; 2 Department of Vascular Neurology, University Hospital Bonn, Bonn, Germany

**Keywords:** CCF, Venous drainage pattern, Uncal veins, ICH

## Abstract

**Background:**

Carotid-cavernous fistulas (CCFs) are abnormal arteriovenous shunts between the carotid arterial system and the cavernous sinus. They most frequently present with ocular symptoms, but intracerebral hemorrhage (ICH) as an initial manifestation is exceedingly rare.

**Case presentation:**

We report the case of a 41-year-old male with a progressive hemiparesis and a spontaneous CCF with atypical reflux into the deep cerebral veins and subsequent intracerebral hemorrhage, discuss the underlying pathomechanism, and provide an overview of the existing literature.

**Conclusion:**

CCFs should be included in the differential diagnosis of ICH, particularly when atypical venous drainage patterns are involved.

## Introduction

Carotid-cavernous fistulas (CCFs) are pathological communications between the carotid arterial system — either the internal (ICA) or external carotid artery (ECA) — and the cavernous sinus. Various classifications were proposed, including direct versus indirect (dural) types, high- versus low-flow dynamics, and traumatic versus spontaneous etiology. Typically, CCFs present with ocular symptoms due to anterior drainage into ophthalmic veins. However, intracerebral hemorrhage (ICH) can be the initial or sole manifestation in rare cases and is related to cortical or deep venous reflux.

This report details a case of a spontaneous CCF presenting atypically with ICH and delayed ocular symptoms, highlighting the critical role of venous drainage patterns in symptomatology and treatment planning.

### Case presentation

A 41-year-old male presented with a 3-week history of progressive right-sided hemiparesis. Upon admission, neurological examination revealed right facial palsy, right hemiparesis, dysarthria and right-sided hemihypesthesia. The initial NIHSS score was 7.

Apart from a history of migraine, no other comorbidities were reported. Until the onset of symptoms, the patient had not experienced any additional complaints, especially no visual symptoms such as double vision.

**Fig. 1:**
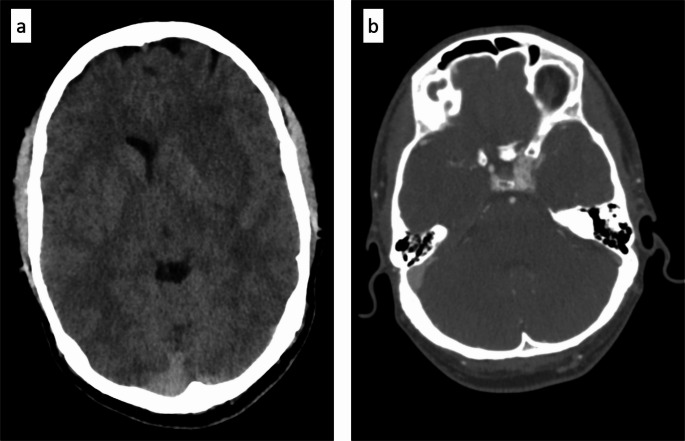
NECT at admission showed a mainly hypodense lesion located at the left lentiform nucleus and external capsula (**a**). CTA showed increased contrast in the left cavernous sinus (**b**)

**Fig. 2:**
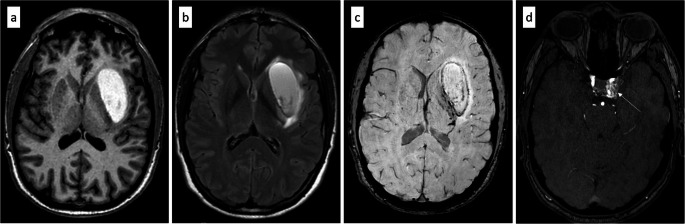
MRI demonstrates a lesion with imaging characteristics consistent with a late subacute hemorrhage in the region of the left external capsule, showing T1- and FLAIR-hyperintense internal signal (**a**, **b**), perifocal edema (b), and peripheral hemosiderin deposition on SWI (**c**). TOF imaging demonstrates arterialized flow signal within the left cavernous sinus (arrow in **d**)

**Fig. 3:**
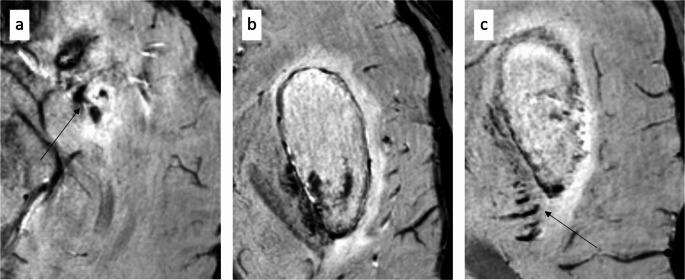
Enlarged SWI sections demonstrating congested uncal veins (**a**), the hemorrhage within the external capsule (**b**), and engorged transmedullary veins cranial to the hemorrhage (**c**)

**Fig. 4:**
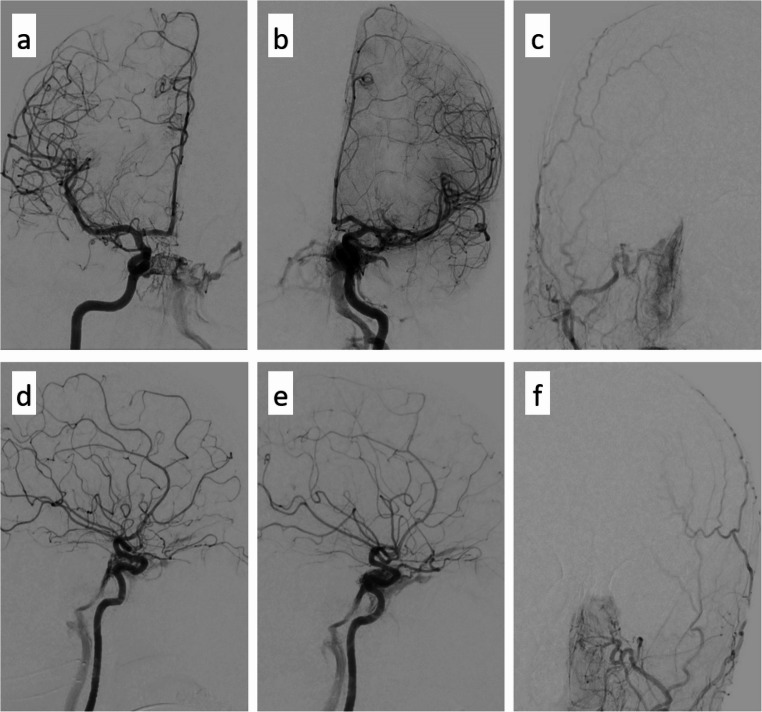
DSA confirmed the presence of a CCF, presumably supplied indirectly by both internal carotid arteries; no arterial inflow from the external carotid artery territory. (**a**, **d**: right ICA AP/lateral; **b**, **e**: left ICA AP/lateral; **c**: right ECA AP; **f**: left ECA AP)

The initial non-contrast CT (NECT) demonstrated a primarily hypodense lesion involving the left lentiform nucleus and external capsule, with faint hyperdense components in the dorsomedial portions. CT angiography (CTA) raised the suspicion of a left-sided CCF (Fig. 1). Subsequent MRI revealed the lesion to be iso- to mildly hyperintense on T2-weighted images, with markedly hyperintense signal on FLAIR and native T1-weighted sequences. Susceptibility-weighted imaging (SWI) revealed predominantly peripheral hemosiderin deposition with largely hyperintense signal characteristics (Fig. 2). Overall, the lesion exhibited imaging features consistent with a late subacute hemorrhage, which correlates with the anamnestic report of progressively worsening symptoms over a period of three weeks. In addition, there was no evidence of extensive venous congestion; however, SWI demonstrated congested uncinate veins caudal to the hemorrhage and engorged transmedullary veins cranial to it (Fig. 3). In keeping with the suspicion of a CCF on CTA, time-of-flight (TOF) imaging showed arterialized flow within the left cavernous sinus (Fig. 2). DSA revealed an indirect, spontaneous Barrow type B carotid-cavernous fistula involving dural branches of both left and right ICA, with predominant posterior and cortical venous drainage, including the uncal veins and the sphenoparietal sinus (Fig. 4). Despite the lack of ocular symptoms there was also drainage into the left ophthalmic veins.

**Fig. 5:**
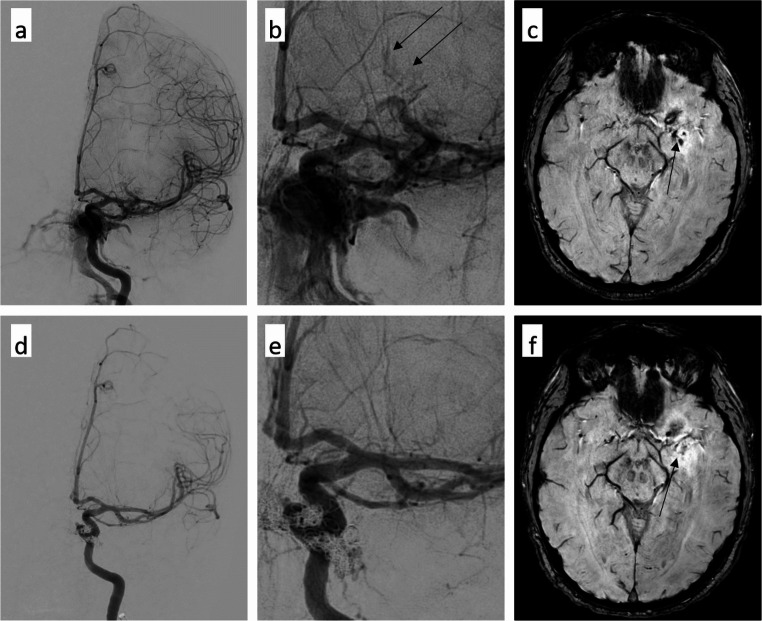
Comparison of pre-interventional (**a**-**c**) and post-interventional images (**d**-**f**) with a focus on congested uncinated veins (arrows in a-c), which are no longer detectable after closure of the CCF (arrow in f)

Transvenous coil embolization was then planned in intubation. In detail, a 7 F guiding catheter (Envoy, Johnson&Johnson) was advanced into the left internal jugular vein via a femoral access, followed by superselective catheterization of the cavernous sinus using a microwire (Synchro-14, Stryker) and microcatheter system (Excelsior-SL 10, Stryker). The fistula was successfully occluded with 14 platinum coils (Target, Stryker). This technique remains first-line for indirect CCFs with accessible venous pouches and avoids the need for transarterial access. Arterial access was only used to run control angiograms. On final run no AV-shunt was visible and postprocedural control MR Imaging 3 days later excluded uncal venous reflux (Fig. 5).

The patient’s post-procedural NIHSS improved to 3, mRS at discharge was 2. No complications were reported.

In the follow-up examination after 3 months, the symptoms had completely regressed. The mRS improved to 0 after rehabilitation.

## Discussion

### Pathophysiology and classification

Carotid-cavernous fistulas are abnormal arteriovenous shunts between the internal (ICA) or external carotid artery (ECA) and the cavernous sinus. The Barrow classification distinguishes four types, with type B involving indirect meningeal feeders from the ICA [1]. While the arterial angioarchitecture determines the Barrow classification, the venous drainage pattern is responsible for venous congestions and potentially hemorrhage [2].

The drainage pathways of a carotid-cavernous fistula can be classified into six distinct types. The most common forms of venous drainage include the anterior drainage into the superior and inferior ophthalmic veins, as well as the posteroinferior drainage into the inferior petrosal sinus, the basilar plexus, and the pterygoid plexus. The posterior drainage into the superior petrosal sinus, the cortical drainage into the sphenoparietal sinus, and the cerebellar drainage into the petrosal vein are less frequent. Additionally, deep drainage can occur through the middle cerebral vein and uncinate veins [3].

While the most prevalent type of drainage, the anterior drainage, typically leads to the classic clinical presentation of a CCF—characterized by primarily ocular symptoms such as pulsatile exophthalmos, chemosis, reduced visual acuity, and elevated intraocular pressure—rarer drainage pathways are associated with a broader range of neurological symptoms. These can include cranial nerve deficits, venous infarctions, ataxia, and congestion-related hemorrhages [4–6].

Various approaches have been proposed to classify carotid-cavernous fistulas based on the characteristics of their venous drainage. For example, Thomas et al. have published a classification system grounded on venous drainage patterns. This classification offers improved correlation with clinical symptoms, angiographic accessibility, and treatment outcomes [6]. According to this system, our patient’s lesion qualifies as Type IV, characterized by retrograde cortical venous drainage, which carries a high risk for intracerebral hemorrhage (ICH) [6].

### Intracerebral hemorrhage due to CCF

While ocular signs such as pulsatile exophthalmos, chemosis, and cranial nerve palsies are the most frequent manifestations of CCFs, intracerebral hemorrhage is a rare and potentially devastating complication. It only occurs in up to 2.6% of cases and may be delayed or serve as the initial presentation, as in this case [5, 7]. Only a few cases of ICH related to CCF without ocular symptoms were reported so far [8].

Pathophysiological, the increased pressure in the cavernous sinus can result in flow reversal with arterialization of the draining veins, venous congestion with edema and finally rupture of the veins with subsequent hemorrhage. Fujita et al. demonstrated that hemorrhage occurs when venous pressure exceeds the structural limits of the venous wall, resulting in vessel rupture [9, 10].

The extent of parenchymal damage and subsequently the extent of clinical symptoms depend on how long the venous reflux can be compensated. Just like arteries, the venous system possesses extensive collateral pathways, allowing for compensation of impaired venous outflow, particularly in the early stages. Initially, the functional impairment of the cells occurs and is later followed by an irreversible injury [9].

This observation is supported by more recent analyses by Alahmari et al., who reported that venous infarction occurs in only 12% of cases associated with cerebral venous sinus thrombosis, whereas vasogenic edema is observed in 66% of cases. Additionally, they described transient diffusion-weighted imaging abnormalities in 8% of patients [10].

Li et al. reported an emergent case of CCF presenting as ICH that required urgent endovascular intervention, highlighting the importance of early recognition and aggressive management [7]. Bram et al. described a similar case where a traumatic CCF led to delayed ICH in a patient with an enucleated orbit, illustrating that even low-flow fistulas can lead to hemorrhage when drainage involves cortical or deep veins [11]. In addition, the study by Bram et al. provides a detailed compilation of all cases reported to date in which a CCF resulted in intracerebral hemorrhage. As demonstrated therein, intracranial hemorrhage typically occurred secondary after other symptoms, most commonly ocular manifestations [11].

The uncal vein, which connects the basal vein of Rosenthal and the cavernous sinus belongs to the deep cerebral veins and is part of the anastomotic venous circle of the base of the brain [12]. It is a known route for pathological venous reflux with subsequent venous congestion in temporal and insular regions, as observed in our case [12]. This is caused by venous reflux from the uncal vein into the inferior striate veins [13]. Ide et al. explored anatomical variations in this venous pathway and their clinical implications in CCFs, illustrating that such patterns may predispose to ICH [14]. This corresponds to Aralasmak et al. who analyzed venous drainage patterns in a cohort of CCFs and confirmed that drainage into cortical or deep medullary veins significantly increased the risk of hemorrhagic and ischemic complications [2].

The venous drainage pattern is not only important for the clinical presentation but also for the treatment strategy. In a technical review of endovascular approaches, Ayman et al. pointed out the importance of understanding venous reflux in CCFs, particularly when planning embolization. Inadvertent occlusion or embolic reflux into cortical veins may worsen the clinical course or precipitate secondary hemorrhage [4]. The underlying pathophysiology is analogous to dural arteriovenous fistulas (dAVFs) with cortical venous reflux. Both conditions share the same mechanism of venous congestions and rupture of small transcerebral veins; this can cause parenchymal hemorrhage without arterial rupture [15].

### Management

Treatment strategies of any dural AV fistula must always be tailored based on both the arterial and venous angioarchitecture. When cortical venous reflux is present, urgent intervention is mandated due to the elevated risk of rebleeding and progressive neurological deterioration [4].

Our patient’s post-procedural NIHSS improved rapidly from 7 to 3, demonstrating partial neurological recovery, most likely after venous reflux and congestion was eliminated. At discharge the mRS was 2. The final outcome depends on the extent of pre-existing hemorrhage, venous congestion, and time to treatment. In our case the 3-month follow-up examination revealed further improvement to an mRS of 0 after rehabilitation.

## Conclusion

This case illustrates the broad clinical variability in the presentation of CCFs and emphasizes that clinical symptoms are determined less by the arterial supply of the fistula and more significantly by the pattern of venous drainage. It represents one of the rare instances in which a CCF manifested as an intracerebral hemorrhage due to venous reflux into the uncal vein, without accompanying ocular symptoms.

## Data Availability

No datasets were generated or analysed during the current study.
